# Supplementary material for: Feeding activity of Eisenia andrei and Enchytraeus albidus under different soil moisture regimes assessed by the bait-lamina test
Source: PLoS One. 2026 Apr 2;21(4):e0328342. doi: 10.1371/journal.pone.0328342 (PMC13046264; doi:10.1371/journal.pone.0328342)
Supplement: S1 File — (DOCX) [file pone.0328342.s001.docx]

S1 File. Supporting Information to

Feeding activity of *Eisenia andrei* and *Enchytraeus albidus* under different soil moisture regimes assessed by the bait-lamina test

Gilda Dell’Ambrogio^1,#a*^, Sophie Campiche^1,#b^, Janine W.Y. Wong^1^, Mathieu Renaud^1^; Christina Lüthi^1^, Inge Werner ^2^, Benoit J.D. Ferrari^1,2^

^1^ Swiss Centre for Applied Ecotoxicology, Lausanne, Vaud, Switzerland

^2^ Swiss Centre for Applied Ecotoxicology, Dübendorf, Zürich, Switzerland

^#a^Current address: Environmental Analytics, Methods Development and Analytics, Agroscope Reckenholz, Zürich, Switzerland

^#b^Current address: EnviBioSoil, Gollion, Vaud, Switzerland

*Corresponding author

# Table of contents

[S1 Table. Field abundances of earthworms for arable soils in Switzerland and neighboring countries 3](#_Toc223686254)

[S2 Table. Field abundances of enchytraeids for arable soils in Switzerland and neighboring countries 4](#_Toc223686255)

[S3 Table. Soil moisture content, feeding activity and mortality data for bait-lamina tests on earthworms and enchytraeids 5](#_Toc223686256)

[S4 Figure. Comparison of nominal and measured soil moisture contents for bait-lamina tests with earthworms in the first (A) and second (B) test 7](#_Toc223686257)

[S5 Figure. Comparison of nominal and measured soil moisture contents for bait-lamina tests with enchytraeids 9](#_Toc223686258)

[References 10](#_Toc223686259)

# S1 Table. Field abundances of earthworms for arable soils in Switzerland and neighboring countries

| Country | Management | Abundance (ind/m^2^) | Source |
| --- | --- | --- | --- |
| Switzerland | undisturbed culture | 100-200 | [1] |
|  | perennial grassland | 200-400 |  |
|  | extensive field | 120-250 | [2] |
|  | natural meadow | 200-300 |  |
|  | extensive meadow | 400-500 |  |

# S2 Table. Field abundances of enchytraeids for arable soils in Switzerland and neighboring countries

| Country | Management | Land use | Abundance (ind/m2) | Source |
| --- | --- | --- | --- | --- |
| Germany | mixed/not specified | crop and grassland sites | 14,000-20,000 | [3] |
|  | mixed/not specified | grassland sites | 13,834 ± 11,312 |  |
|  | mixed/not specified | species-poor intensive grassland on moist sites; 6 sites | 12,480 ± 8,476 |  |
|  | mixed/not specified | trampled grass and park lawns; 7 sites | 13,168 ± 11,347 |  |
|  | mean of mostly undisturbed practices (conservation tillage and shallow conventional tillage, both with no compaction) | Crop (summer barley and winter wheat) | 8,664 | [4] |
|  |  |  | 8,265 |  |
|  | Conservation tillage | Crop (maize) | 9,500 | [5] |
|  | Conservation tillage | Crop (winter wheat) | 6,000 |  |
|  | treated with pesticide, reference non treated and controls together | grassland | 4,000-35,000 | [6] |
|  | mixed/not specified | grassland sites | 2,700-49,000 | Didden 1993. Cited in [6] |
|  | mixed/not specified |  |  |  |
|  | shallow conventional tillage, unwheeled | not specified | ~5,000 (same year of tillage) | [7] |
|  |  |  | ~10,000 (1 year after tillage) |  |
|  |  |  | ~12,000 (2 years after tillage) |  |
| France | organic (no chemical inputs or fertilizers) | Crop | 9,015 | [8] |
|  | integrated system (significant reduction of chemical input and a ploughing every two years) | Crop | 2,903 |  |
| Switzerland | Organic + 44m3/ha slurry in two rates | Crop | 22,000 | [9] |
|  | Organic + 30m3/ha slurry only once | Crop | 27,000 |  |

# S3 Table. Soil moisture content, feeding activity and mortality data for bait-lamina tests on earthworms and enchytraeids

| Nominal relative moisture content (% WHC) | **Measured relative moisture content (% WHC ± SD) at test start** | **Measured relative moisture content (% WHC ± SD) at test end** | **Mean moisture content used for data analysis (% WHC)** | Mean gravimetric moisture content (%) | Mortality rate (%) | Overall feeding activity (% ± SD) | Daily feeding activity (% per day ± SD) |
| --- | --- | --- | --- | --- | --- | --- | --- |
| Earthworms – Test I | | | | | | | |
| **20** | 17.76 **±** 1.33 | **14.64 ± 0.19** | **16** | **7** | n.d. | 0.00 ± 0.00 | 0.00 ± 0.00 |
| **40** | **38.43 ± 0.81** | **34.61 ± 0.37** | **37** | **16** | n.d. | 14.38 ± 5.95 | 7.19 ± 2.97 |
| **60** | **58.20 ± 0.57** | **48.68 ± 6.31** | *53* **(52)** | **24** | n.d. | *41.63 ± 24.68* (43.63 ± 0.39) | *20.82 ± 12.34* (21.82 ± 10.41) |
| **80** | **78.65 ± 0.36** | **87.15 ± 2.18 ^a^** | **79** | 35 | n.d. | 13.25 ± 10.41 | 6.63 ± 5.20 |
| **100** | **95.28 ± 0.79** | **87.33 ± 1.22** | **91** | 41 | n.d. | 22.88 ± 9 .29 | 11.44 ± 4.64 |
| Earthworms – Test II | | | | | | | |
| **30** | **25.60 ± 0.46** | **23.60 ± 0.14** | **25** | 11 | 12 | 3.25 ± 3.46 | 1.63 ± 1.73 |
| **45** | **40.50 ± 0.84** | **38.08 ± 0.22** | **39** | 18 | 4 | 20.63 ± 15.07 | 10.32 ± 7.53 |
| **60** | **50.71 ± 0.54** | **51.80 ± 3.24** | *51* **(52)** | **23** | 0 | *45.63 ± 17.79* (43.63 ± 0.39) | *22.82 ± 9.90* (21.82 ± 10.41) |
| **75** | **71.60 ± 1.03** | **75.49 ± 0.80** | **74** | 33 | 0 | 14.13 ± 12.51 | 7.07 ± 5.75 |
| **90** | **88.39 ± 1.60** | **92.61 ± 4.68** | **90** | **41** | 4 | 4.62 ± 5.92 | 2.32 ± 2.96 |
| **105** | **101.10 ± 2.60** | **106.51 ± 7.16** | **104** | **47** | 0 | 18.50 ± 12.12 | 9.25 ± 6.06 |
| **60 (Ctrl)** | **56.31 ± 2.61** | **n.d.** | **-** | **25** | - | 0.00 ± 0.00 | 0.00 ± 0.00 |
| **100 (Ctrl)** | **n.d.** | **n.d.** | **-** | **-** | - | 2.08 ± 2.60 | 1.04 ± 1.30 |
| Enchytraeids | | | | | | | |
| **40** | **40.67 ± 0.36** | **39.65 ± 0.81** | **40** | 18 | 4 | 44.69 ± 13.29 | 3.72 ± 1.11 |
| **40 (Ctrl)** | **40.67 ± 0.36** | **37.26 ± 0.14** | **-** | - | - | 0.00 ± 0.00 | 0.00 ± 0.00 |
| **50** | **48.83 ± 0.49** | **49.15 ± 1.11** | **49** | 22 | 3 | 76.41 ± 16.79 | 6.37 ± 1.40 |
| **50 (Ctrl)** | **48.83 ± 0.49** | **44.26 ± 0.47** | **-** | - | - | 0.00 ± 0.00 | 0.00 ± 0.00 |
| **60** | **58.61 ± 0.40** | **57.64 ± 0.35** | **58** | 27 | 2 | 84.84 ± 9.26 | 7.07 ± 0.77 |
| **60 (Ctrl)** | **58.61 ± 0.40** | **58.46 ± 0.22** | **-** | - | - | 0.00 ± 0.00 | 0.00 ± 0.00 |
| **70** | **69.10 ± 13.23** | **68.05 ± 1.17** | **68** | 31 | 9 | 91.46 ± 4.69 | 7.62 ± 0.39 |
| **70 (Ctrl)** | **69.10 ± 13.23** | **67.16 ± 1.31** | **-** | - | - | 0.00 ± 0.00 | 0.00 ± 0.00 |
| **80** | **77.45 ± 1.03** | **77.61 ± 1.77** | **78** | 36 | 6 | 3.91 ± 2.90 | 00.33 ± 0.24 |
| **80 (Ctrl)** | **77.45 ± 1.03** | **74.68 ± 0.29** | **~~-~~** | - | - | 0.05 ± 0.10 | 0.08 ± 0.04 |
| ^a^ Considered as outlier and not used to calculate the mean: instead, the value at test start was used for the data analysis. | | | | | | | |

Nominal and measured values of relative soil moisture content at test start and test end expressed as percentage of the maximum water holding capacity (% WHC ± standard deviation, n = 2 for nominal 60 % WHC Earthworms – Test I at test end, for 75 % WHC Earthworms - test II at test start, and for 90 % WHC Earthworms - test II at test end, n = 3 for all other replicates); values of soil moisture content used for the data analysis (mean between test start and test end) expressed as 1) % WHC and 2) percentage of soil dry weight (gravimetric moisture content, %); mortality rate (mean between all replicates, in %); and feeding activity, expressed as 1) percentage of consumed bait at the test end (overall feeding activity) and 2) normalized to test duration (daily feeding activity). Grey shaded lines indicate the reference moisture conditions considered for the tests; n.d. = not determined; Ctrl = negative controls without organisms. Italic values of mean moisture content, overall feeding activity and daily feeding activity refer to the two reference moisture treatments chosen for the two earthworm tests that were pooled, for which the mean value between the two (in brackets) was used for data analysis.

# S4 Figure. Comparison of nominal and measured soil moisture contents for bait-lamina tests with earthworms in the first (A) and second (B) test


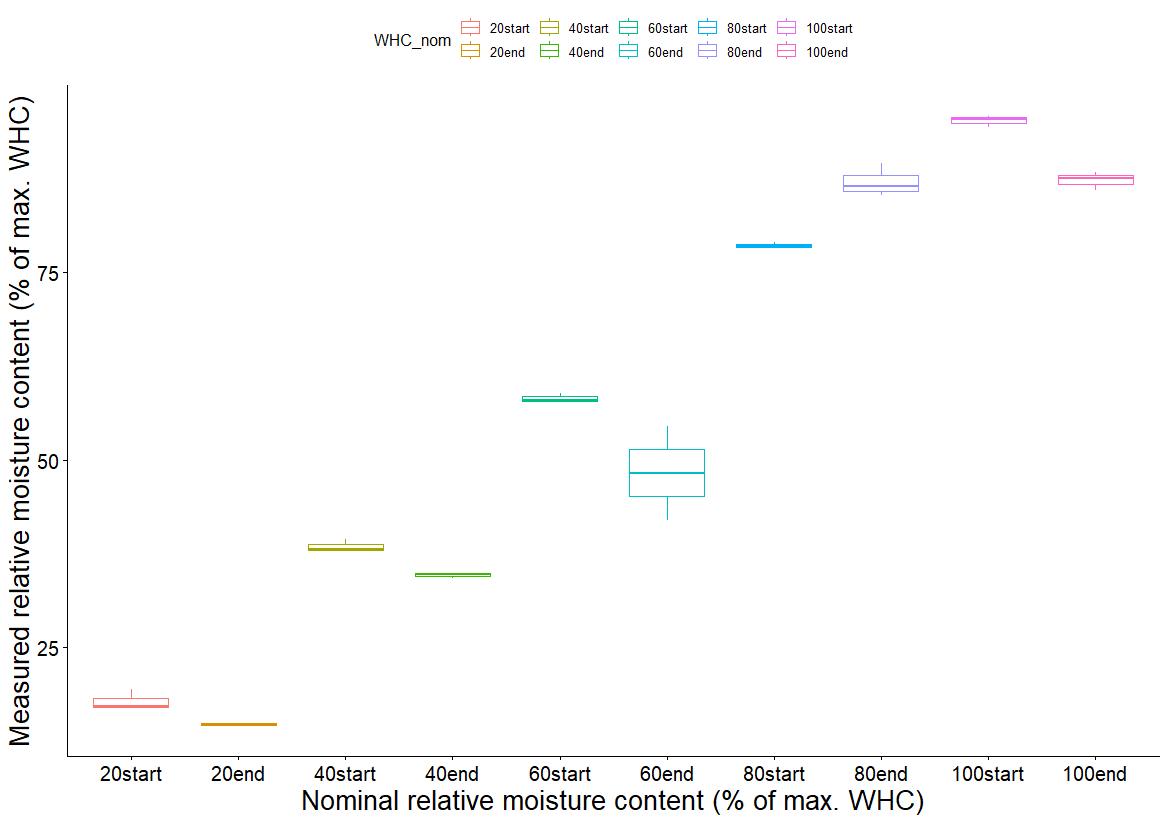
A) Measured relative soil moisture contents (expressed as percentage of the maximum water holding capacity, WHC, n = 2 for nominal 60 % WHC test end, n = 3 for all other replicates) at the test start and test end, compared to the nominal relative soil moisture content (% of WHC) for the first earthworm test.


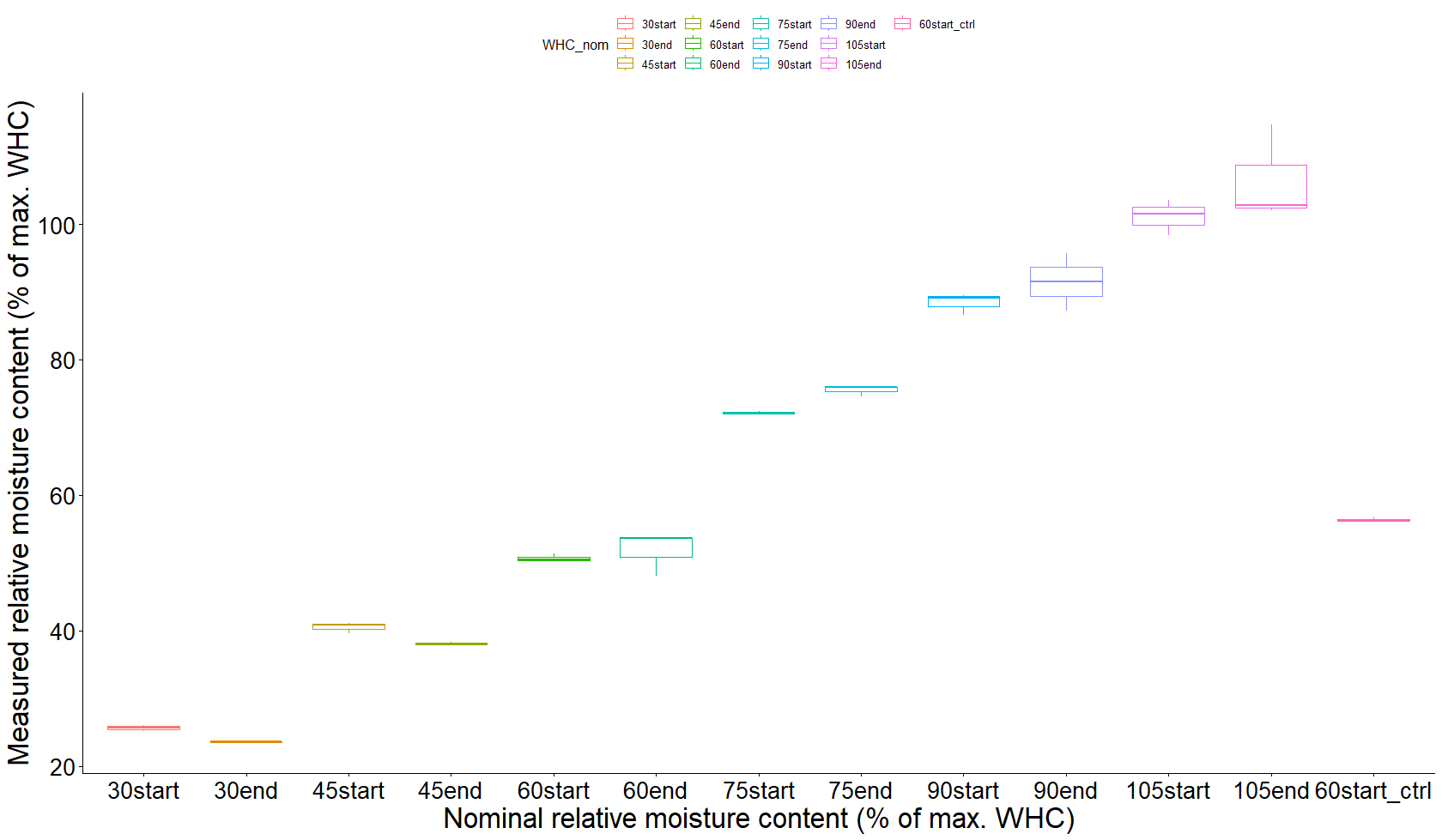
B) Measured relative soil moisture contents (expressed as percentage of the maximum water holding capacity, WHC, n = 2 for nominal 75 % WHC test start and nominal 90 % WHC test end, n = 3 for all other replicates) at the test start and test end, compared to the nominal relative soil moisture content (% of WHC) for the second earthworm test.

# S5 Figure. Comparison of nominal and measured soil moisture contents for bait-lamina tests with enchytraeids


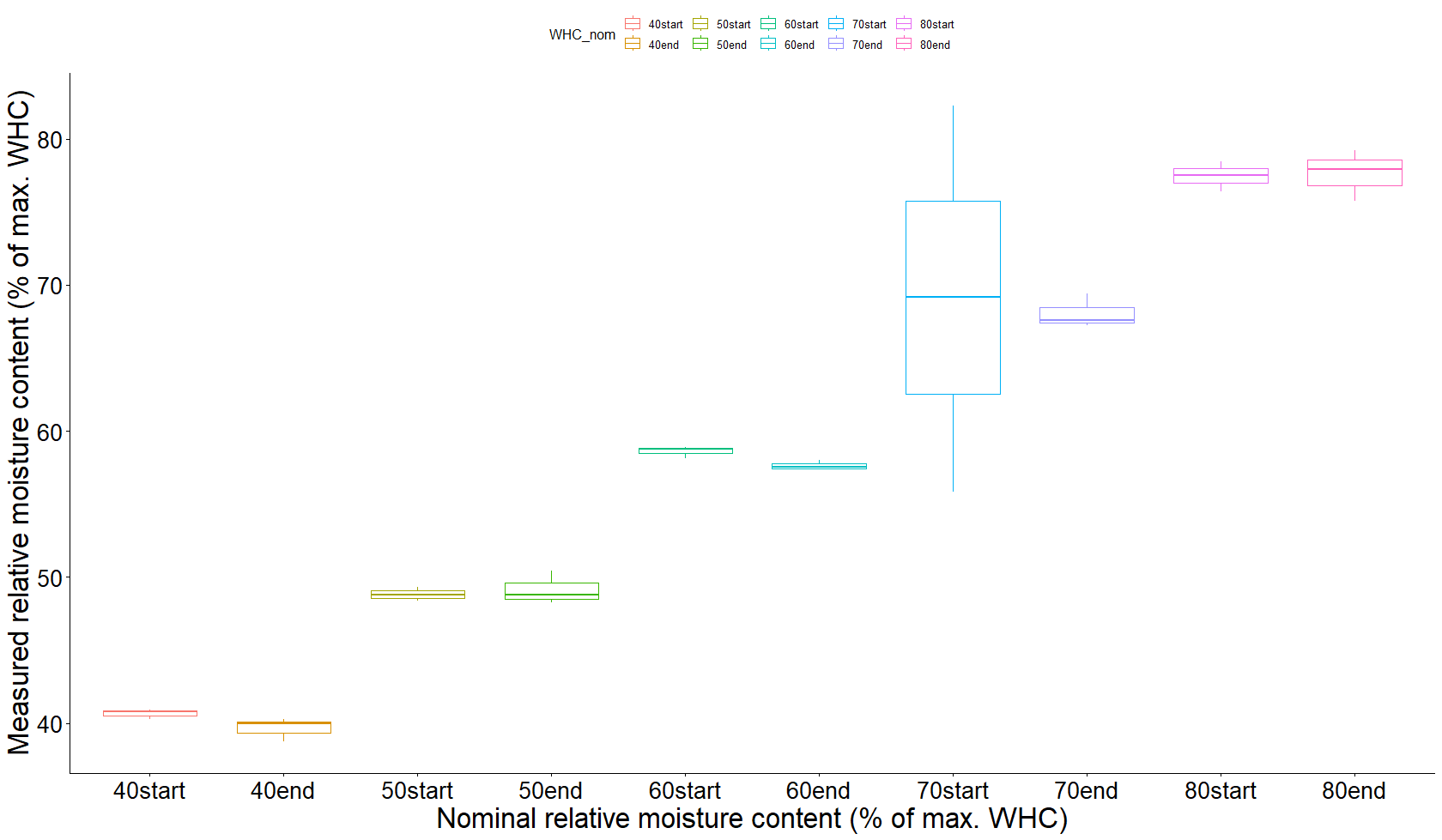
Measured relative soil moisture contents (expressed as percentage of the maximum water holding capacity, % of WHC, n = 3) at the test start and test end, compared to the nominal relative soil moisture content (% of WHC) for the enchytraeid test.

# References

1. Jossi W, Weibel FP, Tamm L, Willer H, (2012) Bénévoles invisibles.
2. FiBL (2013) Regenwürmer—Baumeister fruchtbarer Böden. Frick: Forschungsinstitut für biologischen Landbau (FiBL).
3. Römbke J, Römbke F, Ruf A, Miko L, Schadler M (2013) State of knowledge of enchytraeid communities in German soils as a basis for biological soil quality assessment. Soil Organisms 85(2): 123–146.
4. Röhrig R, Langmaack M, Schrader S (1998) Tillage systems and soil compaction—Their impact on abundance and vertical distribution of Enchytraeidae. Soil and Tillage Research 46(1–2): 117–127.
5. van Capelle C, Schrader S, Brunotte J (2012) Tillage-induced changes in the functional diversity of soil biota—A review with a focus on German data. European Journal of Soil Biology 50: 165–181. https://doi.org/10.1016/j.ejsobi.2012.02.005
6. Römbke J, Schmelz RM, Knaebe S (2009) Field studies for the assessment of pesticides with soil mesofauna, in particular enchytraeids, mites, and nematodes: Design and first results. Soil Organisms 81(2): 237–264.
7. Langmaack M, Wiermann C, Schrader S (1999) Interrelation between soil physical properties and Enchytraeidae abundances following a single soil compaction in arable land. Journal of Plant Nutrition and Soil Science 162(5): 517–525. https://doi.org/10.1002/(SICI)1522-2624(199910)162:5<517::AID-JPLN517>3.0.CO;2-3
8. Ricci F, Coja T, Sattler T, Imfeld G, Schirmel J (2015) Positive effects of alternative cropping systems on terrestrial arthropods. Soil Organisms 87: 71–83.
9. Birkhofer K, Bezemer TM, Bloem J, Bonkowski M, Christensen S, Dubois D, et al. (2008) Long-term organic farming fosters below and aboveground biota: Implications for soil quality, biological control and productivity. Soil Biology and Biochemistry 40(9): 2297–2308. https://doi.org/10.1016/j.soilbio.2008.05.007
