# Supplementary material for: Feeding activity of Eisenia andrei and Enchytraeus albidus under different soil moisture regimes assessed by the bait-lamina test
Source: PLoS One. 2026 Apr 2;21(4):e0328342. doi: 10.1371/journal.pone.0328342 (PMC13046264; doi:10.1371/journal.pone.0328342)
Supplement: Attachment — Submitted filename: Response to Reviewers.docx [file pone.0328342.s002.docx]

We thank the academic editor and the reviewers for their constructive comments. Below, we provide a detailed point-by-point response. Editor and reviewer comments are shown in italics, followed by our responses.

Journal Requirements

*When submitting your revision, we need you to address these additional requirements.*

*1. Please ensure that your manuscript meets PLOS ONE's style requirements, including those for file naming. The PLOS ONE style templates can be found at* [*https://journals.plos.org/plosone/s/file?id=wjVg/PLOSOne_formatting_sample_main_body.pdf*](https://journals.plos.org/plosone/s/file?id=wjVg/PLOSOne_formatting_sample_main_body.pdf) *and* [*https://journals.plos.org/plosone/s/file?id=ba62/PLOSOne_formatting_sample_title_authors_affiliations.pdf*](https://journals.plos.org/plosone/s/file?id=ba62/PLOSOne_formatting_sample_title_authors_affiliations.pdf)

The manuscript style has been adjusted according to the journal requirements.

*2. Please amend your list of authors on the manuscript to ensure that each author is linked to an affiliation. Authors’ affiliations should reflect the institution where the work was done (if authors moved subsequently, you can also list the new affiliation stating “current affiliation:….” as necessary).*

The list of authors has been verified accordingly.

*3. We note that you have included the phrase “data not shown” in your manuscript. Unfortunately, this does not meet our data sharing requirements. PLOS does not permit references to inaccessible data. We require that authors provide all relevant data within the paper, Supporting Information files, or in an acceptable, public repository. Please add a citation to support this phrase or upload the data that corresponds with these findings to a stable repository (such as Figshare or Dryad) and provide and URLs, DOIs, or accession numbers that may be used to access these data. Or, if the data are not a core part of the research being presented in your study, we ask that you remove the phrase that refers to these data.*

The data not shown refers to pilot studies previously performed to explore the suitability of collembolans, earthworms and enchytraeids for the bait-lamina test. As the collembolans showed no significant contribution to bait consumption (confirming findings of other studies referenced in the introduction), the main experiments were conducted with the two oligochaetes only. The collembola data is not a core part of the research. Therefore, the mention on collembolans has been deleted, and the term “pilot studies” has been replaced with the more general “preliminary research”.

*4. If the reviewer comments include a recommendation to cite specific previously published works, please review and evaluate these publications to determine whether they are relevant and should be cited. There is no requirement to cite these works unless the editor has indicated otherwise.*

Not applicable.

Reviewers' comments

*Reviewer's Responses to Questions*

***Comments to the Author*** *1. Is the manuscript technically sound, and do the data support the conclusions?

The manuscript must describe a technically sound piece of scientific research with data that supports the conclusions. Experiments must have been conducted rigorously, with appropriate controls, replication, and sample sizes. The conclusions must be drawn appropriately based on the data presented.*

*Reviewer #1: No*

*Reviewer #2: Yes*

*2. Has the statistical analysis been performed appropriately and rigorously?*

*Reviewer #1: No*

*Reviewer #2: Yes*

*3. Have the authors made all data underlying the findings in their manuscript fully available?

The* [*PLOS Data policy*](http://track.editorialmanager.com/CL0/http:%2F%2Fwww.plosone.org%2Fstatic%2Fpolicies.action%23sharing/1/010f019864e4e45e-a6c3453a-70a1-4282-80c0-519f533930bd-000000/Qem7NAECCF7bZKilIlFd1m8uwy8RxHUWDpYA5q-jYQw=222) *requires authors to make all data underlying the findings described in their manuscript fully available without restriction, with rare exception (please refer to the Data Availability Statement in the manuscript PDF file). The data should be provided as part of the manuscript or its supporting information, or deposited to a public repository. For example, in addition to summary statistics, the data points behind means, medians and variance measures should be available. If there are restrictions on publicly sharing data—e.g. participant privacy or use of data from a third party—those must be specified.*

*Reviewer #1: Yes*

*Reviewer #2: Yes*

*4. Is the manuscript presented in an intelligible fashion and written in standard English?

PLOS ONE does not copyedit accepted manuscripts, so the language in submitted articles must be clear, correct, and unambiguous. Any typographical or grammatical errors should be corrected at revision, so please note any specific errors here.*

*Reviewer #1: No*

*Reviewer #2: Yes*

*5. Review Comments to the Author

Please use the space provided to explain your answers to the questions above. You may also include additional comments for the author, including concerns about dual publication, research ethics, or publication ethics. (Please upload your review as an attachment if it exceeds 20,000 characters)*

*Reviewer #1: The paper describes a laboratory experiment involving bait-lamina and the effect of soil moisture on the feeding activities of two soil animal species. While the subject matter is potentially interesting for readers, the manuscript cannot be published due to the weak presentation of the data and errors in the statistical analysis.
The most important analysis is missing - to what extent are 52% and 68% for the two species statistically significantly different?*

For earthworms, 52% WHC is significantly different to all the other tested moisture contents, based on ANOVA followed by a Dunnet’s post-hoc test with Holm’s correction.

For enchytraeid, the same analysis revealed no statistical differences between the moisture contents of 49, 58 and 68% WHC, which were, however, significantly different to the other moisture contents tested. The manuscript has been edited to clarify these statistical results, now talking about an optimal range between 49 and 68% for enchytraeids, rather than a single peak of 68%.

The difference in feeding efficiency between the two species was compared in a descriptive manner at the two reference moisture contents (52% and 49% WHC): this was not tested statistically because the two conditions are not directly comparable. All the data on the influence of moisture on feeding activity was subject to statistical analyses.

*The text is too long-winded. The style needs to be changed to be more concise, with excessive words and repetitions removed.*

The text has been reviewed and many paragraphs rephrased to make it more concise.

*It is quite surprising, that earthworms contribute in feeding activity on bait lamina strips – they are rather bigger soil animals. Maybe Authors used very small (young) individuals? Please specify animals age/size. Are you sure that the earthworm treatments have not been “contaminated” with enchytraeids?*
All the individuals used for the tests were adults. The age/size of the organisms used in our experiments is specified in the methods section (see L212-213 in “Revised Manuscript with Track Changes”): “Only adults aged between 2 and 8 months and with an individual wet weight between 300 to 600 mg were used for the tests.”

The contribution of earthworms to bait lamina consumption has also been observed in several other studies cited in the manuscript [4-7,14,15] and was thus expected.

We confirmed that the earthworm treatments were not contaminated with other organisms by running additional treatments without earthworms as additional controls. These controls showed zero to very low consumption of the bait, and the latter was attributed to the high amount of water which could have dissolved the bait material. This information is included on L465-473.

*L163 why two tests? Please combine it and just mark, that experiment was performed in two stages*

This is due to methodological reasons and was done at two steps: first, one test was performed at 20, 40, 60, 80, and 100 % of the WHC. By looking at the results, we observed an interesting trend and we decided to run a second test to better capture the change in feeding activity at intermediate moisture contents, i.e. 30, 45, 60, 75, 90, and 105 % of the WHC. This explanation has been added to the manuscript (L177-181).

*L171 why were the same values not used as for earthworms? Such results do not compare well*

Based on pilot studies, we expected to observe the response of enchytraeids under a narrower range of soil moisture, compared to earthworms. This explanation has been added to the manuscript (L190-193).

*L194 was the biomass of earthworms and enchytraeids comparable? There is difficult to find these information in the text. Moreover, moisture effect may depend on animals density.*

Yes, the biomass used was 2.25 ± 0.75 g for earthworms and 2.30 ± 0.69 g for enchytraeids and we consider these two values sufficiently comparable. We highlighted this point in the methods (L222-224).

We recognize that the animal densities are different between organisms, but this is part of the limitations of the experimental design, and we chose biomass as the best parameter for comparison of the two species. Moreover, both the tested biomass and densities of the two organisms were carefully selected so that they would best reflect field conditions and allow for data interpretation at a realistic scale.

*L224 why was it done? Rinsighted strips can wash out the medium, making such a measurement pointless*

The strips were rinsed to remove any excess of soil particles. The rinsing is recommended in key references [3,7], which are cited in the manuscript. We have confirmed in previous experiments [16, 20] that this does not affect the results. We consider that this statement is well supported by the cited references and does not require additional changes.

*Tab.1 I do not see the point of stating these values. Moreover, table is too big and unclear*

The journal requires all the raw data to be available and the table supports the discussion on the test performance. However, we agree that the table is too complex and some information may be unnecessary. The table has therefore been deleted and the relevant information moved to the supplementary information and merged with an already existing table (S3).

*L259 combining two species in this analysis has no sense, they do not interact with each other*

Although the two species were tested in two different experiments and they didn’t interact in the biological sense, we consider that from a statistical point of view it does make sense to test for interaction. This interaction indicates whether the moisture effect of feeding activity is different depending on the species. The inclusion of the interaction term in the regression analysis is then crucial to explore whether the feeding activities of the two species respond differently to changes in soil moisture, which was actually the case as our data shows.

*Reviewer #2: The article submitted by Dell’Ambrogio et al. addresses a technique—the use of Bait Lamina—that provides a simple and low-cost method for assessing soil functional quality. As such, it represents a promising tool for evaluating the functional impacts of various environmental stressors on this key ecosystem compartment. However, its use remains relatively limited, leading to a knowledge gap that sometimes hampers the interpretation of results. In this context, the study by Dell’Ambrogio et al. appears both relevant and important for improving the robustness and applicability of future research employing this technique.

The study, conducted exclusively under laboratory conditions using a single soil type and two model organisms, appears to have been carried out in a rigorous manner, following standardized protocols (number of tested conditions, number of replicates, number of individuals, etc.) that allow for robust statistical analysis and ensure the scientific reliability of the results. The findings are original and respond to a clearly identified knowledge need.
Therefore, this article may be considered suitable for publication in PLOS ONE.

However, I question the relevance of so strongly associating this study with the aim of improving in situ use of bait lamina. The experimental conditions implemented in this study are quite distant from those encountered in field situations, both in terms of the organisms present and the physico-chemical properties of the soils (which are not sieved in situ and may span a wide range of pedological conditions). The authors are aware of this and have discussed it in the manuscript. Nonetheless, I believe that the stated objective of the study should be more aligned with the actual scope of the results obtained. In other words, there is no need to overstate the findings, which are valuable in their own right even without a direct connection to field conditions.
For example, I am personally not convinced by the comparison made between the present study’s results and those from previous in situ work by Campiche et al. (Fig. 4). In my view, linking these two sets of results is too speculative—despite the tempting similarity in trends observed with E. albidus—given the many differences between the two studies (which, again, are well discussed by the authors). In my opinion, this comparison should be used solely to feed the discussion, especially to highlight perspectives and the need for further research to better interpret in situ responses. However, if the authors decide to maintain this comparison, it seems essential to provide more information about the data from Campiche et al., to better assess to what extent those results are generalizable across diverse environmental conditions.*

We understand the general concern and agree that similarity between the models for enchytraeids and the field should not be interpreted as a major influence of the enchytraeid community in the field. We agree that this kind of hypothesis appears too speculative. Still, we would like to keep the comparison between the models to stimulate the discussion and show that trends can be similar under a comparable moisture range. The text in the manuscript has been adapted accordingly (L636-663).

*Beyond this general comment, which reflects my main concern about the current manuscript, here are a few additional—mostly minor—comments that may help improve the clarity and quality of the manuscript:
________________________________________
Abstract
• In light of the above comment, I recommend revising the abstract to reduce the emphasis on in situ applications.*

The last sentence of the abstract has been rephrased accordingly (L41-46).

*Highlights
• Similarly, the final bullet point seems too speculative to be featured as a highlight.
• Also, the phrase “field situation” is too vague and should be clarified or removed.
• Some highlights appear too long—please verify that they meet the journal’s character limits.*
The bullet points have been rephrased/deleted accordingly (L53-61).

*Introduction
• L.70–71: The word “soil” appears too frequently in the same sentence—please try to avoid this repetition.*

The word soil has been removed once (L64-65).

*• L.94: Replace “at the Ecotox Centre” with “Campiche et al.”*

Done (L91-92).

*• L.95–97: The information provided here about the work by Campiche et al. is insufficient to allow the reader to fully assess its scope or the generalizability of its findings.*

More information on the study have been added (L91-100).

*Materials & Methods
• L.161–162: Avoid future tense (“will be expressed”).*

Changed to present tense (L175).

*• L.163–175: Why is the number of replicates different between the two biological models? This would be helpful to clarify for readers.*

The workload was more intense for collecting and inserting the enchytraeids. An explanation has been added (L193-195).

*• L.220: The phrase “once a week” is odd given that the test lasted only 12 days. Consider rephrasing as “at most once.”*

The formulation has been replaced with “periodically” (L259).

*Results
• L.265: How can the high mortality rate observed in earthworms at 25% WHC be explained? Unless I missed it, this does not appear to be discussed.*

The high mortality at this treatment is likely due to the fact that the soil was too dry for the earthworms. At such extremely low soil moisture, some mortality is to be expected, but what is important is that the mortality was low in the reference moisture contents. We added this explanation (L310-312).

*• Table 1: What does the number 4 in the final column of the last row refer to?*

This was a mistake. We thank the reviewer for having noticed it and we corrected it in the table.

*• Figures 3 & 4: Rather than showing only the means, would it not be more informative to display all replicates to better convey data variability?*

The figures have been changed as suggested.

*• Figure 4: How many replicates were considered to calculate the means from Campiche et al.? Were these replicates all from the same soil or different soils? Such information is largely missing in the current integration of this prior work.*

The number of replicates has been added in the figure caption. More information on the study from Campiche et al. has been added in the introduction (L91-100).

*Discussion
• L.370–371: The explanation that lower variation in moisture in the enchytraeid test is due to moisture adjustments is not very convincing, especially considering the difference in test duration (2 vs. 12 days).*

We added another possible explanation (L451-453): “This difference might be due to higher metabolic water requirements of earthworms, which could have contributed to greater soil moisture loss”.

*• L.416: What exactly is meant by “interaction within soil communities,” and how could this influence bait lamina feeding? This point should be expanded.*

We added the following information (L514-517): “Finally, interactions with other soil organisms, such as other mesofauna or microbial communities, may further influence earthworm feeding behavior, for example through resource competition or microbial-mediated changes in nutrient availability.”

*• L.433: The question of soil quantity should be discussed in terms of density per unit of soil. Were densities higher in the studies cited compared to the present one? Has this hypothesis been tested in previous research?*

When comparing in terms of density of individuals, the studies are not very comparable. We expanded that point and suggested another possible reason for the differences, highlighting the fact that a comparison is difficult (L537-544): “Even after accounting for this normalization, the studies are difficult to compare because different densities of organisms per unit of soil were used. Interestingly, density alone doesn’t seem to fully explain the observed differences: the study with the highest density [29] reported feeding rate more comparable to our results whereas in the study with the lowest density a higher bait consumption was observed [13]. One possible reason for this is that in the latter case, the test organisms were distributed across different soil depths, which could have facilitated access to the bait stick”.

*• L.474: While the point about “sandy loamy soils” is valid, what about other soil types? This should be addressed to better define the study’s limitations.*

This limitation has been added to the text (L607-608).

*• L.484–485: Could mobility also be a biological factor explaining the differences in bait lamina feeding efficiency?*

Yes, it can be related to the difference in the spatial scale of the two functional roles. We thank you the reviewer for the suggestion and added this hypothesis to the text (L621-623).

*• L.498–500: Due to the significant differences between lab and field conditions (unsieved soil, natural species different from lab models, etc.), this hypothesis appears too speculative in its current form. The same applies to lines 521–522 and 541–544.*

The hypotheses have been removed (L639-641, L666-668, L694-695).

*6. PLOS authors have the option to publish the peer review history of their article (*[*what does this mean?*](https://track.editorialmanager.com/CL0/https:%2F%2Fjournals.plos.org%2Fplosone%2Fs%2Feditorial-and-peer-review-process%23loc-peer-review-history/1/010f019864e4e45e-a6c3453a-70a1-4282-80c0-519f533930bd-000000/cpCFA46V2hTRbFF-mqTjjB-nRk5xdWrYVTqWeez81C8=222)*). If published, this will include your full peer review and any attached files.


If you choose “no”, your identity will remain anonymous but your review may still be made public.*


***Do you want your identity to be public for this peer review?*** *For information about this choice, including consent withdrawal, please see our* [*Privacy Policy*](https://track.editorialmanager.com/CL0/https:%2F%2Fwww.plos.org%2Fprivacy-policy/1/010f019864e4e45e-a6c3453a-70a1-4282-80c0-519f533930bd-000000/2Ty587ynK8lwMfJ6QWaMcGB-28KEcm6fW_UPwP8o2-E=222)*.*

*Reviewer #1: No*

*Reviewer #2: No*
